# A large‐scale, multicenter characterization of *BRAF* G469V/A‐mutant non‐small cell lung cancer

**DOI:** 10.1002/cam4.7305

**Published:** 2024-05-21

**Authors:** Han Wu, Jian Feng, Shun Lu, Jia Huang

**Affiliations:** ^1^ Department of Surgical Oncology, Shanghai Lung Cancer Center, Shanghai Chest Hospital Shanghai Jiao Tong University School of Medicine Shanghai China; ^2^ Department of Thoracic surgery, Shanghai Chest Hospital Shanghai Jiao Tong University Shanghai China; ^3^ Department of Medical Oncology, Shanghai Lung Cancer Center, Shanghai Chest Hospital, School of Medicine Shanghai Jiao Tong University Shanghai China

**Keywords:** *BRAF*, G469, gefitinib, non‐small cell lung cancer (NSCLC), targeted therapy

## Abstract

**Background:**

Mutated *BRAF* is identified in 1%–5% non‐small cell lung cancer (NSCLC) patients, with non‐V600 mutations accounting for 50%–70% of these. The most common non‐V600 mutation is *BRAF* G469V/A. Currently, there are no targeted therapies available for non‐V600 mutated patients. A recent report provided interesting preclinical evidence revealing sensitivity of *BRAF* G469V to epidermal growth factor receptor (EGFR) inhibitors, raising the possibility of repurposing anti‐EGFR agents. It is therefore worthy to characterize the clinical and molecular features of *BRAF* G469V/A‐mutant NSCLC to provide more insights for precision therapy.

**Methods:**

We conducted a retrospective screening of 25,694 Chinese patients with advanced or metastatic NSCLC to identify individuals with mutated *BRAF*. Additionally, we performed similar screenings on patients with adenocarcinoma (LUAD) from The Cancer Genome Atlas (TCGA) cohort (*n* = 567) and the MSKCC cohort (*n* = 1152). Subsequently, we characterized the clinical and molecular features of the patients carrying *BRAF* mutations.

**Results:**

*BRAF* G469V was identified in 28 (0.1%) patients from the Chinese NSCLC cohort and 5 (0.9%) from TCGA‐LUAD. Notably, none was identified in the MSKCC cohort. G469A was found in 79 (0.3%) Chinese patients, 2 (0.4%) from TCGA‐LUAD, and 9 (0.8%) from the MSKCC cohort. Relative allele frequency analysis suggested most *BRAF* mutations as driven clones. Tumor mutation burden (median 4 mutations/Mb) was not significantly different between patients carrying G469V, G469A, V600E, or other *BRAF* mutations. Surprisingly, *KRAS* mutations were found in approximately 50% of patients with G469V mutation and about 8% of patients with G469A mutation, representing a prominent potential resistance mechanism against EGFR inhibitors. Structural modeling suggested *BRAF* G469V and G469A as binding partners of gefitinib.

**Conclusion:**

Our large‐scale analysis characterized the prevalence and mutational landscape of *BRAF* G469V/A‐mutant NSCLC and proposed gefitinib as a potential option, providing a basis for further investigations on treating *BRAF*‐mutated NSCLC.

## 1 INTRODUCTION

Lung cancer represents a severe, global burden on public health. Approximately 85% lung cancer cases are non‐small cell lung cancer (NSCLC),^1^ of which 1%–5% are *BRAF*‐mutated.^2^ *BRAF* encodes a serine–threonine protein kinase pivotal in transducing signals from RAS to the MAPK pathways, which ultimately promote cell proliferation and survival.^3^ Mutations on the V600 codon account for 30%–50% of somatic *BRAF* mutations in NSCLC.^4^ Targeted therapies involving dabrafenib combined with trametinib and encorafenib combined with binimetinib have received approval from the US Food and Drug Administration for the treatment of metastatic NSCLC with *BRAF* V600E mutations.^5^ G469V and G469A are the most common Class II mutants and function as RAS‐independent dimers.^6^ However, there are currently no targeted treatments available.^2^ A major focus of ongoing drug development initiatives is the identification of selective RAF dimer inhibitors against cancers driven by Class II *BRAF* mutations.^5^

Huo et al. recently reported unexpected sensitivity of *BRAF* G469V‐mutant preclinical models to gefitinib and osimertinib, two tyrosine kinase inhibitor (TKIs) targeting mutant epidermal growth factor receptor (EGFR).^4^ In a lung adenocarcinoma patient‐derived xenograft model and a cell line derived from the xenograft, *BRAF* G469V was the only known oncogenic mutation, and knockdown of *BRAF* and not *EGFR* killed the xenograft‐derived cell line. In vitro binding experiments using purified G469V‐mutant *BRAF* suggested direct binding with the EGFR inhibitors, which were further supported by structural modeling.^4^ However, a pooled analysis of 40 patients with *BRAF* non‐V600‐mutant metastatic colorectal cancer revealed rare response to therapy containing anti‐EGFR antibody treatment in carriers of Class II mutations, including two patients with G469V and four with G469A mutations.^7^

These findings raised the interesting possibility of repurposing anti‐EGFR agents for targeting *BRAF* G469V/A and along with more questions to be answered, such as the prevalence, clinical characteristics, and concurrent therapeutic vulnerabilities of patients harboring this mutation. In this study, we addressed these questions with a large‐scale screening and characterization of *BRAF* G469V/A‐mutant NSCLC.

## 2 METHODS

### 2.1 Patients

We retrospectively screened 25,694 Chinese patients who were diagnosed with advanced or metastatic NSCLC, including 84.3% lung adenocarcinoma, 9.4% lung squamous cell carcinoma, and 6.3% other NSCLC. Genomic profiles were obtained through targeted sequencing using a 168‐ or 520‐gene panels between January 2018 and December 2021 for those harboring mutated *BRAF*. We also obtained data from adenocarcinoma (LUAD) patients from The Cancer Genome Atlas (TCGA; *n* = 567)^8^ and the MSKCC cohort (*n* = 1152)^9^ and screened for *BRAF*‐mutated cases. This study was approved by the Institutional Review Board of Shanghai Chest Hospital (No. KS1735). Informed consent was obtained from all patients or corresponding family members. Clinicopathological features were retrieved from the patients' medical records.

### 2.2 DNA extraction, library construction, targeted sequencing, and bioinformatic analysis

All following procedures were performed at a clinical diagnostic laboratory certified by the College of American Pathologists (CAP) and Clinical Laboratory Improvement Amendments (CLIA). DNA was extracted with a QIAamp DNA tissue kit from formalin‐fixed, paraffin‐embedded tissue samples and with a QIAamp Circulating Nucleic Acid kit (Qiagen, Düsseldorf, Germany) from liquid biopsies. DNA library construction and targeted sequencing with a commercial panel (Burning Rock Biotech, Guangzhou, China) were performed as previously described.^10^, ^11^ Sequencing was performed with a Nextseq500 sequencing (Illumina, San Diego, USA) at target depths of 1000× for tumor samples and 10,000× for liquid biopsy samples.

Bioinformatic processing was performed as previously described.^10^, ^11^ Tumor mutation burden (TMB) was calculated as number of non‐synonymous somatic alterations on the coding regions of the targeted genes per million base pairs after excluding variants with allelic frequency <2% from tissue samples or <0.2% from liquid biopsy samples. Relative allelic frequency (RAF) was defined as the ratio of the allelic frequency of a specific variant to the maximum allelic frequency of all variants detected from a sample.

### 2.3 Structural modeling assay of gefitinib and *BRAF* mutants

To calculate interaction energy between gefitinib and each *BRAF* mutant, the protein sequence of *BRAF* was downloaded from Ensembl (http://www.ensembl.org/index.html). Then, PolyPhen‐2 (http://genetics.bwh.harvard.edu/pph2/index.shtml) was used to search for the most similar 3D visualization of *BRAF*. In this study, we used 4MBJ (*BRAF* structure in PDB) for binding assay. Next, the sequence of *BRAF* wild type (WT), G469V, G469A, and V600E were uploaded to SWISS‐MODEL (https://swissmodel.expasy.org/) for model construction. Furthermore, the 2D structure of gefitinib was downloaded from PubChem (https://pubchem.ncbi.nlm.nih.gov/) for further analysis. At last, SwissDock (http://www.swissdock.ch/docking) and CHIMERA (http://www.cgl.ucsf.edu/chimera/) were used for protein binding interaction simulation and visualization, respectively.

### 2.4 Statistical analyses

Fisher's exact test was used to compare the proportions of values of a categorical variable, and Wilcoxon signed‐rank test was used to compare the central tendency of a continuous variable. All statistical analyses were performed in R (version 4.0.2, The R Foundation, https://www.r‐project.org/). Significance was set at two‐side p < 0.05.

## 3 RESULTS

### 3.1 Genomic cohort characteristics in NSCLC

From January 2018 to December 2021, a total of 26,657 NSCLC samples from 25,694 patients were subjected to targeted DNA sequencing using a panel of 168 or 520 cancer‐associated genes (Burning Rock Biotech, Guangzhou, China). All patients were Chinese and were included in our study for baseline characteristics analysis. Among them, 996 patients were identified with *BRAF* mutation. However, only 853 patients had clinical information on age, gender, and maximum allele frequency (MaxAF). Twenty‐eight of these patients were identified with *BRAF* G469V mutation (3.2%), 79 patients with G469A (9.3%), 235 patients with V600E (27.5%), and 511 patients with other *BRAF* mutation variants (59.9%), which included K601E (8.6%, *n* = 73), D594G (6.1%, *n* = 52), and other canonical mutations. The clinical characteristics of all patients with NSCLC and a subset of patients with lung squamous cell carcinoma are summarized in Table 1.

**TABLE 1:** Baseline characteristics of BRAF mutated NSCLC patients (*n* = 853).

All NSCLC patients						
Clinicopathological feature	Overall (*n* = 853)	G469V (*n* = 28)	G469A (*n* = 79)	V600E (*n* = 235)	Other (*n* = 511)	*p* Value
Age						
Mean (SD)	61.49 (11.24)	60.46 (9.55)	60.85 (10.21)	61.83 (11.17)	61.49 (11.53)	0.65
Median [IQR]	63.00 [55.00, 69.00]	62.00 [52.75, 66.00]	61.00 [56.00, 67.50]	64.00 [54.00, 70.00]	63.00 [54.50, 70.00]	
Gender						
Female	354 (41.50%)	9 (32.14%)	33 (41.77%)	129 (54.89%)	183 (35.81%)	<0.001
Male	499 (58.50%)	19 (67.86%)	46 (58.23%)	106 (45.11%)	328 (64.19%)	

The subset of patients with LUSC						
Clinicopathological feature	Overall (*n* = 35)	G469V (*n* = 0)	G469A (*n* = 3)	V600E (*n* = 2)	Other (*n* = 30)	*p* Value
Age						
Mean (SD)	67.69 (8.89)	–	66.00 (4.36)	72.00 (0.00)	67.57 (9.47)	0.758
Median [IQR]	69.00 [62.00, 73.00]	–	68.00 [64.50, 68.50]	72.00 [72.00, 72.00]	69.50 [61.50, 73.75]	
Gender						
Female	4 (11.4)	–	0 (0.0)	2 (100.0)	2 (6.7)	NA
Male	31 (88.6)	–	3 (100.0)	0 (0.0)	28 (93.3)	

### 3.2 Incidence of *BRAF* mutations in different populations

**FIGURE 1:**
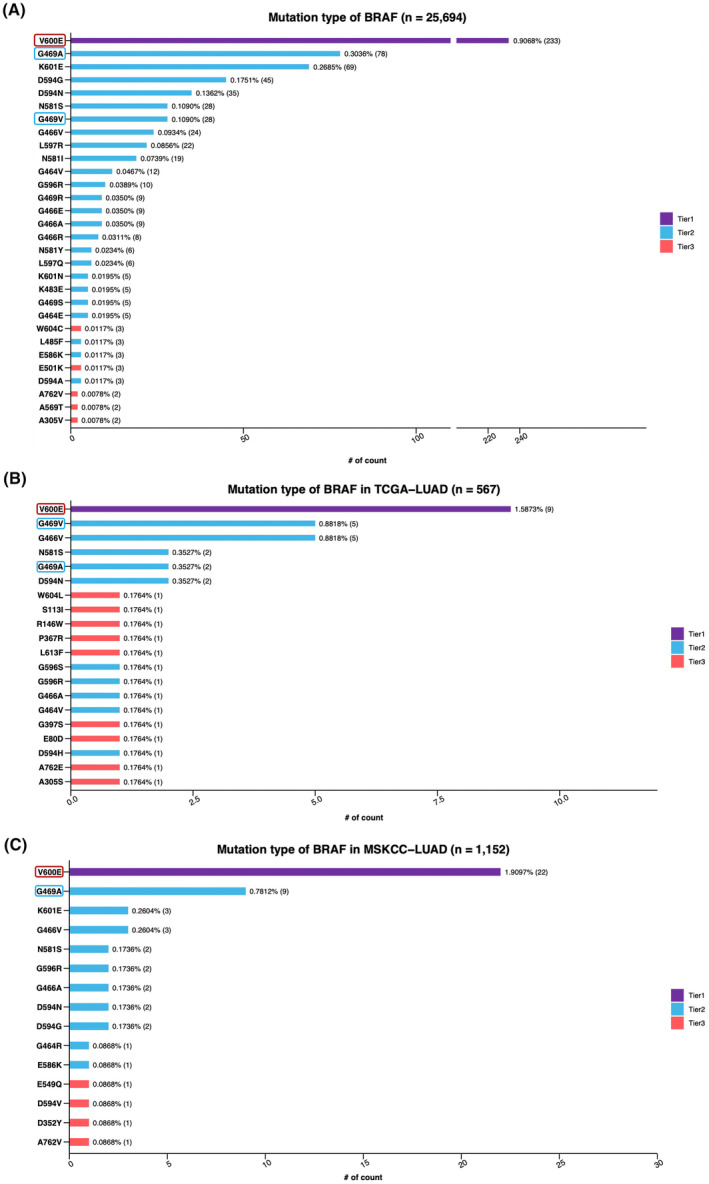
Distribution of *BRAF* mutation in three cohorts. (A) Chinese non‐small cell lung cancer patients. (B) TCGA‐LUAD database. (C) MSKCC database.

We then characterized the mutational landscape of *BRAF* G469V/A‐mutant NSCLC from three datasets (Chinese patients, TCGA‐LUAD, and MSKCC). The *BRAF* variants and prevalence rates are shown in Figure 1.

We first analyzed multiple mutation variants of *BRAF* in Chinese NSCLC patients. A total of 28 patients harbored the G469V mutation, which was present in ~0.1% of all patients. Another variant on the G469 genomics locus, G469A, was present in ~0.3% (*n* = 78) patients. The most common of *BRAF* mutations was V600E, which was detected in ~0.9% cases (*n* = 233; Figure 1A). These results suggest that the patients who had *BRAF* G469V/A are very rare in the Chinese population. However, these patients should be treated with specific therapies.

To further calculate the population frequency of *BRAF* G469V mutation in the TCGA‐LUAD cohort (Figure 1B), we observed that approximately 0.88% (*n* = 5) patients (*n* = 567) were identified with *BRAF* G469V mutation. On the other hand, G469A was only found in approximately 0.35% (*n* = 2) patients in the cohort. As expected, the largest subgroup was *BRAF* V600E, which accounted for approximately 1.6% (*n* = 9) of the LUAD cohort, which was consistent with previous reports.^12^, ^13^, ^14^

To detect the various distribution of *BRAF* mutations, we also calculated the frequency of *BRAF* alterations in the MSKCC database (*n* = 1152). Unfortunately, no patient was identified with the G469V mutation (Figure 1C). As a result, the frequencies of G469A and V600E were approximately 0.78% (*n* = 9) and 1.91% (*n* = 22), respectively.

Together, these findings confirmed that the frequencies of *BRAF* alterations, especially of G469V/A, were diverse in different populations. This further indicated that it was a challenge to treat *BRAF* G469V/A‐mutant patients.

### 3.3 Variant clonality analysis of *BRAF* mutations in NSCLC

**FIGURE 2:**
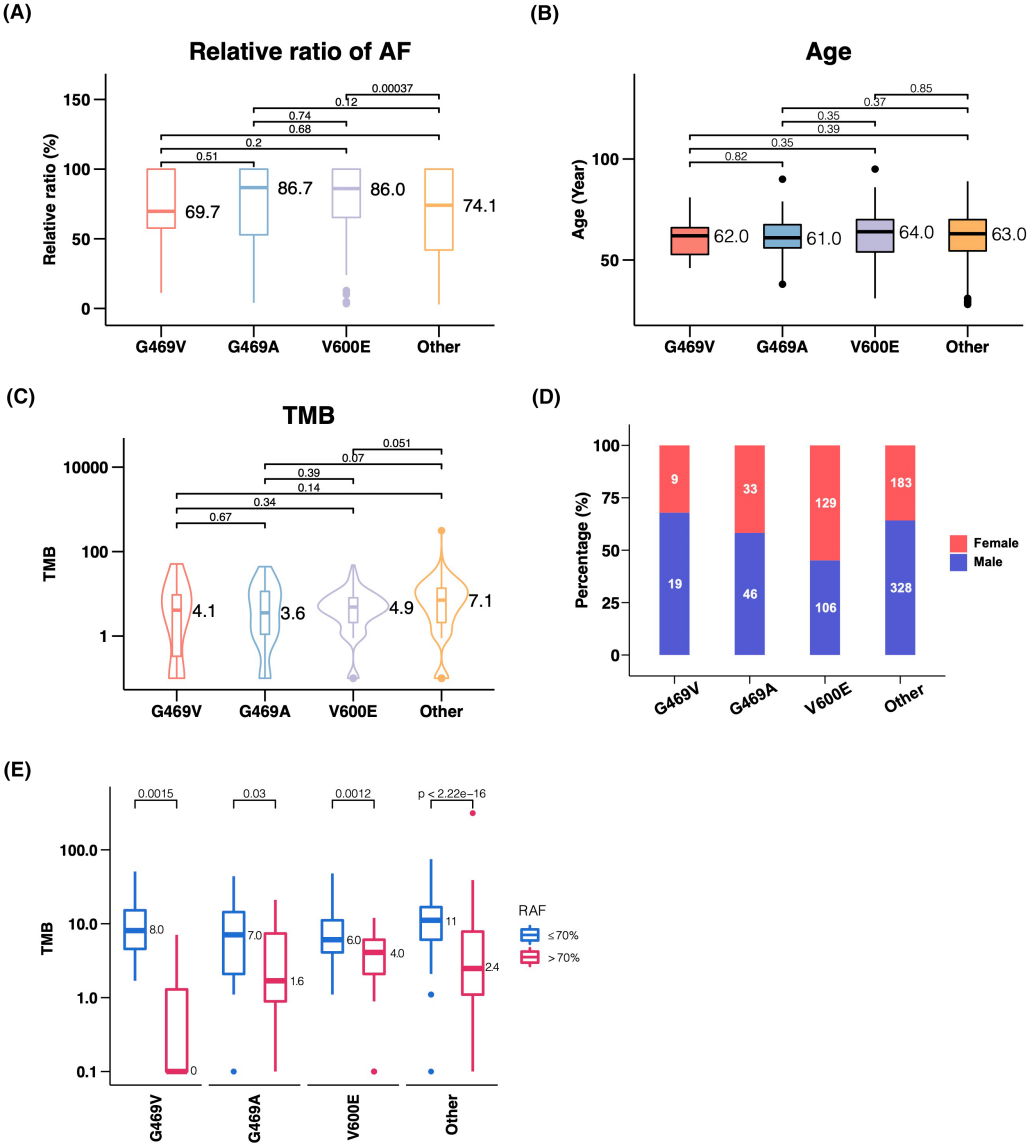
Relative allele frequency (RAF) (A), age (B), tumor mutation burden (TMB) (C), and gender (D) in lung cancer patients carrying different *BRAF* mutations. (E) Boxplot showing the TMB of two groups (RAF >70% and ≤70%). The median value of TMB was labeled.

**FIGURE 3:**
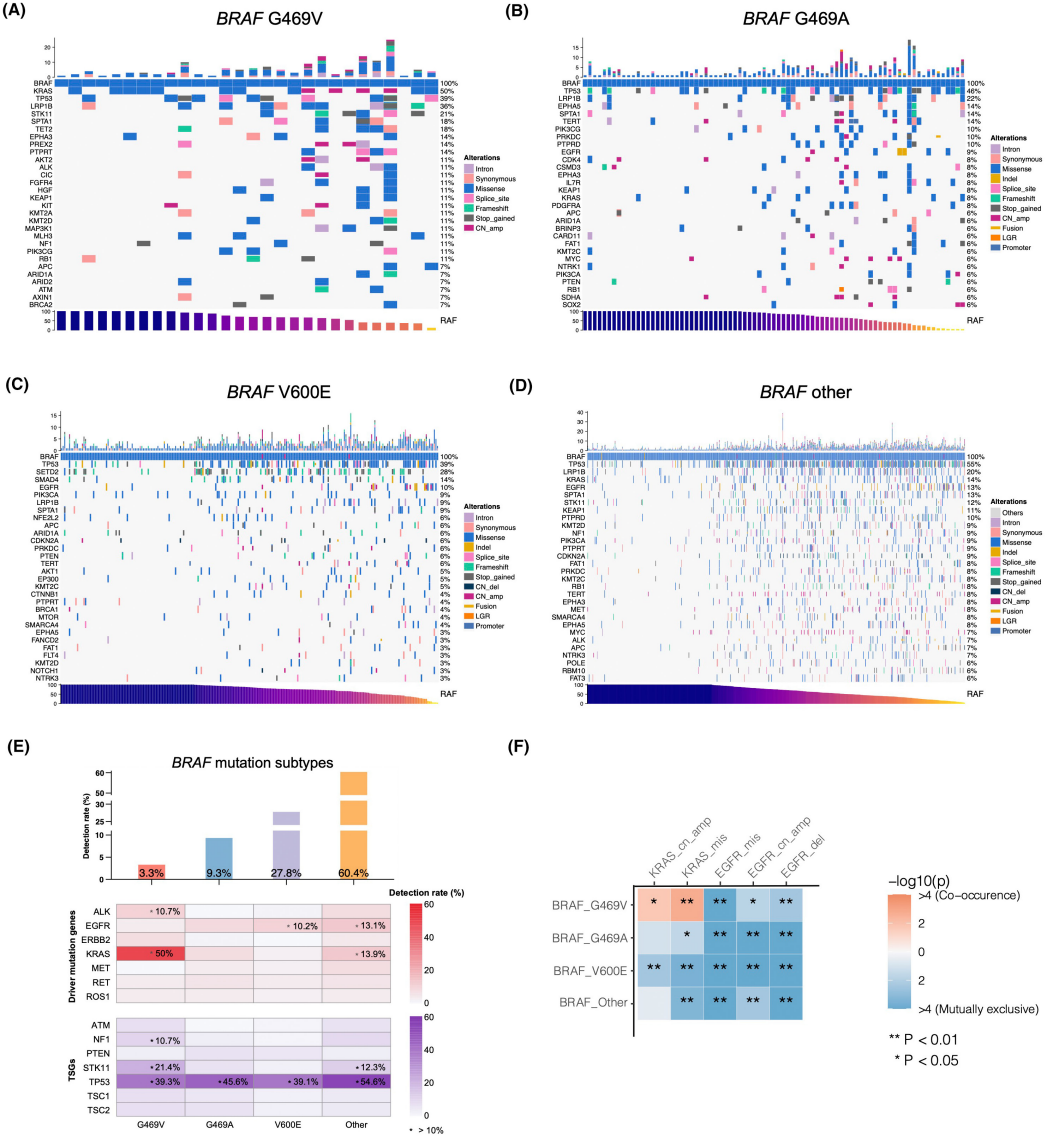
An oncoprint summarizing the mutational landscape of lung cancer patients carrying *BRAF* G469V (A), G469A (B), V600E (C), and other mutations (D). (E) Bar plots at the top illustrate the distribution of detection rate for each group. Heatmaps in the middle and at the bottom summarize the frequencies of altered oncogenic drivers and tumor suppressor genes (TSGs) in patients who harbored the indicated mutant. (F) A heatmap of concurrent and mutually exclusive mutations in *BRAF*‐mutated patients.

To further evaluate dominant versus nondominant clonal relationship of different gene variants, we then introduced the relative abundance of different allele frequency patterns. Briefly, variant allele frequency (VAF) of each variant and MaxAF of patient were calculated, then VAF was divided by MaxAF in the given patient and defined as RAF for each variant. As a result, RAFs could represent genetic variations that reveal the clonality for each mutation. To examine the clonal dominance of *BRAF* mutations, distribution of RAF is shown in Figure 2A. The median RAFs for G469V, G469A, V600E, and other *BRAF* mutations were 69.7%, 86.7%, 86.0%, and 74.1%, respectively. High RAF (*BRAF* mutation AF/MaxAF) indicates that these *BRAF* mutations may drive tumor evolution, so we defined these mutations with RAF > 70% as *BRAF*‐driven clones. We found that the median TMB value was 8.0 muts/Mb in cases with G469V and RAF > 70%, while it was 0 in cases with RAF≤70% (Figure 2E). Furthermore, we observed the majority of patients exhibited fewer mutations in *BRAF*‐driven clones (RAF >70%, Figures 2E and 3A–D), especially in the G469V group (Figure 3A). These results indicated that *BRAF* mutations may be a potential driver of NSCLC.

### 3.4 Clinical information analysis of *BRAF* mutations in NSCLC

We observed no significant difference in the age distribution among patients carrying various *BRAF* variants, with a median age of 62 years (Figure 2B). Then, tumor mutation burden (TMB) was assessed, revealing no statistically significant variance (Figure 2C), with a median TMB of 4 muts/Mb. However, a marked difference was identified between female and male patients with *BRAF* mutations (p < 0.001). Notably, females accounted for 32.14% and 41.77% in the G469V and G469A subgroups, respectively, whereas 54.89% patients were female in V600E (Figure 2D). Together, these findings suggested different gender distribution of each *BRAF* mutation.

### 3.5 Molecular characterization of *BRAF* mutations in NSCLC

To explore the genomic profile of *BRAF*‐mutant patients, we evaluated all patients with NSCLC harboring *BRAF* G469V, G469A, V600, and other mutations. *TP53* mutations were the most common concurrent mutation, observed in 39.3% of individuals with *BRAF* G469V, 45.6% with G469A, 39.1% with V600E, and 54.6% with other *BRAF* mutations (Figure 3A–E). The canonical *EGFR* driver mutations were mutually exclusive from *BRAF* G469V, while approximately 10% patients carried with co‐occurring *EGFR* mutations in *BRAF* V600E (10.2%) and other (13.1%) groups. In addition, 83.9% of these co‐occurring *EGFR* missense mutations are driver pathogenic variants (L858R, T790M, C797X, G719X, S768X, etc.). Unexpectedly, 50% *BRAF* G469V‐mutant and 8% *BRAF* G469A‐mutant patients harbored co‐occurring *KRAS* mutations (Figure 3A,E,F). Among co‐occurring *KRAS* mutations, 89.3% of missense variants are pathogenic variants (G12X/G12X, Q61X, A59X, etc.). These results suggested that the patients with co‐mutations, especially those who harbored concurrent *KRAS* and *BRAF* mutations, may require different treatments.

### 3.6 Molecular modeling of gefitinib and *BRAF* mutants

**FIGURE 4:**
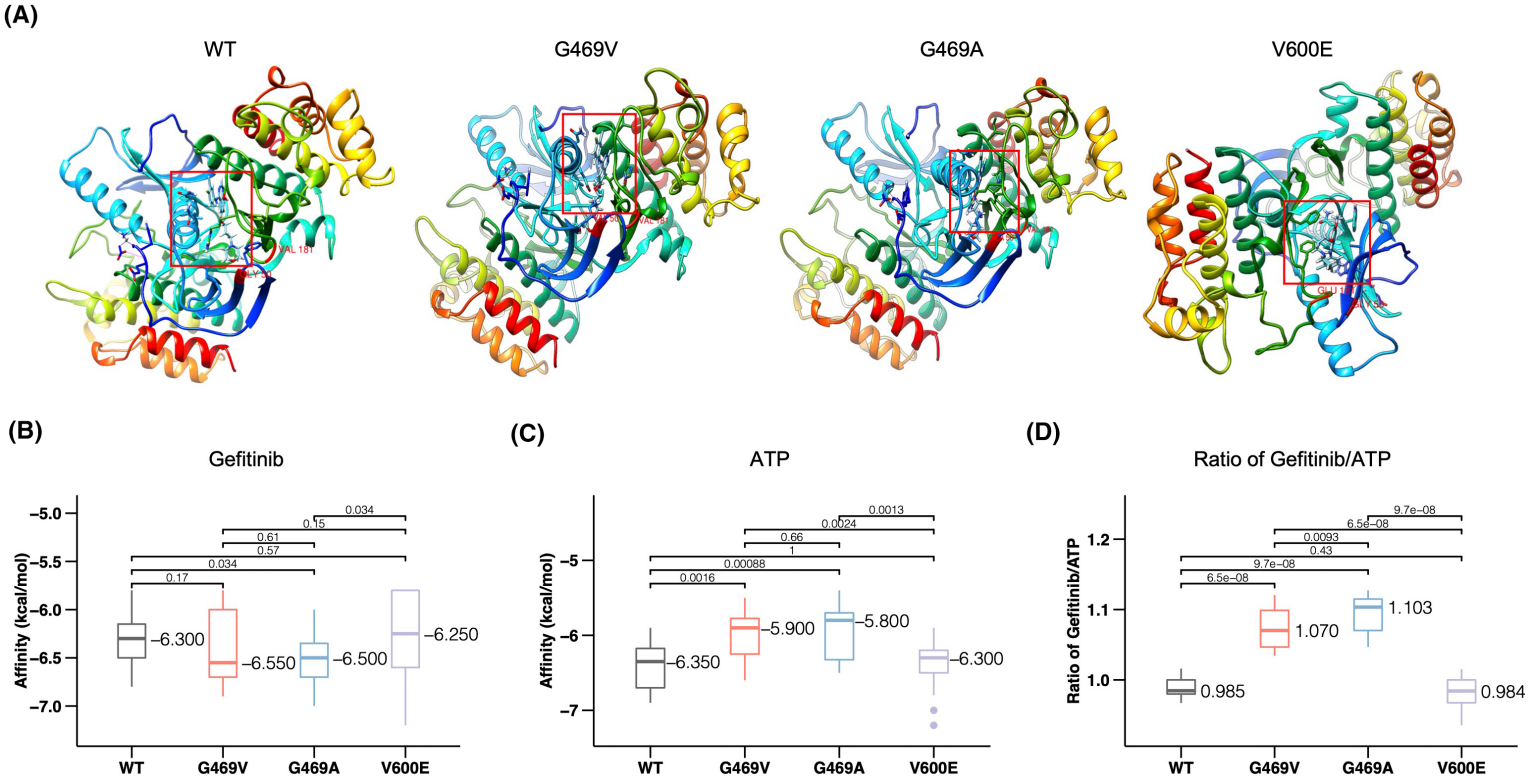
Structural modeling of binding between mutant *BRAF* and EGFR inhibitor gefitinib. (A) Structural similarity between gefitinib and *BRAF* mutants. The binding sites were labeled with red box. Interaction energies were calculated for gefitinib (B) and ATP (C) as shown. (D) Ratios of interaction energies of gefitinib and of ATP with wild type (WT) and mutant *BRAF* were calculated with a bootstrapping approach.

To further explore how gefitinib inhibits *BRAF* variants, we compared the drug‐/ligand‐bound protein structures of *BRAF* and gefitinib. Binding of *BRAF* WT (PDB: 4MBJ), G469V, G469A, or V600E to gefitinib was modeled (Figure 4A). In this binding assay, we found the interaction of *BRAF* G469V with gefitinib was greater (with lower affinity) than that of WT and other *BRAF* mutants (Figure 4B). However, after calculating the interaction energy between ATP and each mutant, we found that both G469V and G469A were reduced (with high interaction energy, Figure 4C). Comparing the interaction energy of gefitinib by ATP, we found G469V and G469A had higher binding interaction with gefitinib than WT and V600E (Figure 4D). This result suggested that the *BRAF* G469V and G469A substitution may facilitate transition of ATP by gefitinib, making both G469V and G469A mutant more sensitive to gefitinib.

## 4 DISCUSSION

Using three large‐scale NSCLC or LUAD cohorts (*n* = 27,413 in total), we identified 853 *BRAF*‐mutated patients, 28 (0.10%) of whom carried G469V and 79 (0.29%) carried G469A, analyzed the demographic, clinical, and molecular features of the G469‐mutated patients, and provided structural modeling evidence supporting direct binding of G469A with gefitinib, an EGFR TKI. Non‐V600E mutations accounted for 59.9% (511 out of 853), and G469 was the most frequently mutated site among these mutations. Molecular analyses revealed high RAF levels of *BRAF* G469A or G469V, suggestive of clonal dominance and high likelihood of being oncogenic drivers, and significant concurrent genetic alterations that may guide therapeutic strategies. For instance, *KRAS* alterations were found in 50% (14 out of 28) *BRAF* G469V‐mutant patients, only 2 of whom carried the now actionable G12C mutation. Considering the potential roles of aberrant *KRAS* in mediating resistance to EGFR TKIs, our findings suggested the need for alternative options for this specific patient subpopulation.

*BRAF* encodes a RAS‐regulated protein kinase in the RAS/MAPK pathway. Upon EGFR activation, *BRAF* relays the signal to downstream MEK, which in turn activates MAPK signaling. *BRAF* mutations are typically grouped into three classes based on effect on kinase activity and RAS dependence, and G469V and G469A both belong to Class II mutations, leading to formation of constitutively active, RAS‐independent dimers.^15^, ^16^ Current evidence is scanty on efficacy of *BRAF* or MEK inhibition in treating *BRAF* G469‐mutant NSCLC, coming mostly from isolated cases and preclinical models.^4^, ^16^, ^17^ Although not entirely consistent, most evidence suggests lack of activity. Negrao et al. reported rapid disease progression in 2 months in a G469V‐mutant LUAD patient on combination therapy with da*braf*enib and trametinib, an MEK inhibitor.^16^ The authors also constructed a G469A‐mutant patient‐derived LUAD cell line and observed resistance to MEK with or without *BRAF* inhibitors. By contrast, Gardini et al. reported an interesting case of synchronous G469V‐mutant NSCLC and *BRAF* WT hepatocellular carcinoma, in which sorafenib elicited partial response of the primary lung lesion, complete response of the he contralateral lung metastasis, and stable disease of the liver lesion.^17^ It is possible that mutational landscape had contributed to the conflicting clinical outcomes in these two cases. Concurrent *APC* R1040fs*16, *CHD2* L1383*, and *NFKBIA* and *NKX2‐1* amplifications were detected in the case from Negrao et al.,^16^ and the second patient was tested for status of *EGFR, KRAS, NRAS, PIK3CA, BRAF, ERBB2, ALK, DDR2, MAP2K1*, and *RET*, in which only *BRAF* was found aberrant.^17^ It is therefore unknown whether other genetic abnormalities, such as those in NF1 (a negative regulator of *BRAF*) and CDK4,^15^ had affected the response to sorafenib. This contrast also highlighted the utility of broad‐panel next‐generation sequencing in gaining molecular insights into drug efficacy and development.

Studies in other cancers may also provide hints on the therapeutic sensitivity of *BRAF* G469‐mutant NSCLC.^7^, ^18^ G469V was predicted as deleterious in an in silico study of skin melanoma, and molecular dynamic simulations suggested that G469V had lower binding energy than the WT variant for da*braf*enib.^18^ While more clinical validation is needed, these results suggested potential resistance to currently available *BRAF* inhibitors and EGFR blockade, thereby highlighting the significance of EGFR TKIs in targeting G469‐mutated *BRAF*. In this study, we revealed a combined prevalence of 0.39% in NSCLC, corresponding approximately to 6000 deaths per year based on the 2020 GLOBOCAN statistics.^19^

Our structural modeling analysis suggested increased affinity to gefitinib for the G469V mutant, which was consistent with Huo et al.,^4^ and for G469A, warranting more preclinical and clinical characterization.

The study is limited by a few factors. In addition to its retrospective nature, our study lacks data on sensitivity of G469‐mutant NSCLC cell models to EGFR TKIs. Considering the high incidence of concurrent *KRAS* alterations in G469V‐mutant patients, future studies may also need to investigate the efficacy of combined inhibition of *KRAS* or downstream MAPK pathway members such as MEK and ERK.

## 5 CONCLUSION

Through a large‐scale screening of 27,413 NSCLC or LUAD patients in total, we identified 28 (0.10%) carriers of *BRAF* G469V and 79 (0.29%) of G469A. Analysis of genomic profiles suggested likely oncogenic driver roles of these mutants. Found in ~50% *BRAF* G469V‐mutant patients, *KRAS* alterations, most of which not G12C, represented a prominent potential resistance mechanism against EGFR TKIs. Structural modeling supported direct binding between EGFR TKI gefitinib and *BRAF* G469V, and suggested *BRAF* G469A as a binding partner. In summary, our study characterized the prevalence and mutational landscape of *BRAF* G469V/A‐mutant NSCLC and proposed gefitinib as a potential treatment option.

## AUTHOR CONTRIBUTIONS

**Han Wu:** Data curation (lead); project administration (lead). **Jian Feng:** Formal analysis (lead); methodology (lead). **Shun Lu:** Conceptualization (equal); writing – original draft (equal); writing – review and editing (equal). **Jia Huang:** Conceptualization (equal); writing – original draft (lead); writing – review and editing (equal).

## FUNDING INFORMATION

None.

## CONFLICT OF INTEREST STATEMENT

This research is sponsored by received research support from AstraZeneca, Hutchison, BMS, Heng Rui, Beigene and Roche, Hansoh, Lilly Suzhou Pharmaceutical Co., Ltd. Received speaker fees from Astra Zeneca, Roche, Hansoh, and Hengrui Therapeutics. An advisor and consultant of Astra Zeneca, Pfizer, Boehringer Ingelheim, Hutchison MediPharma, ZaiLab, GenomiCare, Yuhan Corporation, Menarini, InventisBio Co., Ltd., Shanghai Fosun Pharmaceutical (Group) Co., Ltd., Simcere Zaiming Pharmaceutical Co., Ltd., and Roche.

## ETHICS STATEMENT

All procedures performed in studies involving human participants were in accordance with the ethical standards of the Institutional Review Board of Shanghai Chest Hospital (No. KS1735) and with the 1964 Helsinki Declaration and its later amendments or comparable ethical standards.

## PATIENT CONSENT FOR PUBLICATION

All patients had provided written informed consent for participating in the study.

## Data Availability

The datasets used and/or analyzed during the current study are available from the corresponding author on reasonable request.
